# Supplementary material for: Does Early Childhood Caries Increase Caries Development among School Children and Adolescents? A Systematic Review and Meta-Analysis
Source: Int J Environ Res Public Health. 2022 Oct 18;19(20):13459. doi: 10.3390/ijerph192013459 (PMC9603429; doi:10.3390/ijerph192013459)
Supplement: Supplementary file 1 [file ijerph-19-13459-s001.zip › File S1. Search strategy.pdf]

Supplementary file S1. Search strategy

PubMed
Searched on 28 Jul 2022
Results: 839

((((("Dental Caries"[Mesh]) OR (carie*[Text Word] OR carious[Text Word] OR DMF[Text Word])) OR ((dental[Text Word] OR tooth[Text Word] OR teeth[Text Word] OR enamel[Text Word] OR dentin*[Text Word]) AND (decay*[Text Word] OR cavit*[Text Word] OR demineral i*[Text Word] OR reminerali*[Text Word] OR "white spot*"[Text Word]))) AND ((("Infant"[Mesh]) OR "Child"[Mesh]) OR (child*[Text Word] OR infant*[Text Word]))) AND (("Risk Factors"[Mesh:NoExp]) OR ((risk[Text Word]) AND (factor*[Text Word] OR predictor*[Text Word] OR predicator*[Text Word])))) AND ((("Cohort Studies"[Mesh]) OR "Case-Control Studies"[Mesh]) OR (longitudinal studies[Text Word] OR follow-up studies[Text Word] OR case-control studies[Text Word] OR cohort studies[Text Word] OR prospective studies[Text Word]))

Ovid Medline
Ovid MEDLINE(R) and Epub Ahead of Print, In-Process, In-Data-Review & Other Non-Indexed Citations, Daily and Versions(R) 1946 to February 26, 2021
Searched on 28 Jul 2022
Results: 746

1. exp Dental Caries/
2. (carie* or carious or DMF).tw.
3. ((dental or tooth or teeth or enamel or dentin*) and (decay* or cavit* or demineral i* or reminerali* or "white spot*")).tw.
4. 1 or 2 or 3
5. exp Infant/ or exp Child/
6. (child* or infant*).tw.
7. 5 or 6
8. Risk Factors/
9. (risk and (factor* or predictor* or predicator*)).tw.
10. 8 or 9
11. exp Cohort Studies/ or exp Case-Control Studies/
12. (longitudinal studies or follow-up studies or case-control studies or cohort studies or prospective studies).tw.

13. 11 or 12
14. 4 and 7 and 10 and 13

Embase
Embase Classic+Embase 1947 to 2021 February 26

Searched on 28 Jul 2022
Results: 380

1. exp dental caries/
2. (carie*or carious or DMF).tw.
3. ((dental or tooth or teeth or enamel or dentin*) and (decay* or cavit* or deminerali* or reminerali* or "white spot*")).tw.
4. 1 or 2 or 3
5. exp Infant/ or exp Child/
6. (child* or infant*).tw.
7. 5 or 6
8. Risk Factor/
9. (risk and (factor* or predictor* or predicator*)).tw.
10. 8 or 9
11. exp cohort analysis/ or exp case control study/
12. (longitudinal studies or follow-up studies or case-control studies or cohort studies or prospective studies).tw.
13. 11 or 12
14. 4 and 7 and 10 and 13

Web of Science
Searched on 28 Jul 2022
Results: 633

TS=((dental caries OR carie* OR carious OR DMF) OR ((dental OR tooth OR teeth OR enamel OR dentin*) AND (decay* or cavit* or deminerali* or reminerali* or "white spot*")))
AND TS=(child* OR infant*)
AND TS=(longitudinal studies OR follow-up studies OR case control studies OR cohort studies OR prospective studies)
AND TS=(risk AND (factor* OR predictor* OR predicator*))
CI-SSH, ESCI Timespan=All years
