# Supplementary material for: Does Early Childhood Caries Increase Caries Development among School Children and Adolescents? A Systematic Review and Meta-Analysis
Source: Int J Environ Res Public Health. 2022 Oct 18;19(20):13459. doi: 10.3390/ijerph192013459 (PMC9603429; doi:10.3390/ijerph192013459)
Supplement: Supplementary file 1 [file ijerph-19-13459-s001.zip › File S2. Reasons for exclusion after full text reading.pdf]

Supplementary file S2. Reasons for exclusion after full text reading

	Reasons for exclusion	Year	Title	Journal	Authors
1	No relevant data	2021	Development of dental caries and risk factors between 1 and 7 years of age in areas of high risk for dental caries in Stockholm, Sweden.	Eur Arch Paediatr Dent	M. Anderson; G. Dahllof; A. Warnqvist; M. Grindefjord.
2	No differentiation of caries increment between primary and permanent dentition	2011	Longitudinal associations between children's dental caries and risk factors	J Public Health Dent	O. Chankanka; J. E. Cavanaugh; S. M. Levy; T. A. Marshall; J. J. Warren; B. Broffitt; J. L. Kolker
3	Primary dentition (dmft) increment	2019	Increase in dental caries and change in the socioeconomic profile of families in a child cohort of the primary health care in Northeast Brazil	BMC Oral Health	M. de Melo; W. V. de Souza; P. S. A. de Goes
4	Participants involved children > 71 months	1992	A multivariate model to predict caries increment in Montreal children aged 5 years	Community Dent Health	M. Demers; J. M. Brodeur; C. Mouton; P. L. Simard; L. Trahan; G. Veilleux
5	No relevant data	1992	The University of North Carolina Caries Risk Assessment study: further developments in caries risk prediction	Community Dent Oral Epidemiol	J. A. Disney; R. C. Graves; J. W. Stamm; H. M. Bohannan; J. R. Abernathy; D. D. Zack
6	Tooth level increment	2021	The Impact of Early Childhood Factors on Dental Caries Incidence in First Permanent Molars: A 7-Year Follow-Up Study.	Caries Res.	B Emmanuelli; J. K. Knorst; G. R. Menegazzo; F. M. Mendes; T. M. Ardenghi.
7	No differentiation of caries increment	2012	The natural history of dental caries lesions: a 4-year observational study	J Dent Res	A. Ferreira Zandoná; E. Santiago; G. J. Eckert; B. P. Katz; S. Pereira

	between primary and permanent dentition				de Oliveira; O. R. Capin; M. Mau; D. T. Zero
8	Participants involved children > 71 months	2019	Models to Predict Future Permanent Tooth Caries Incidence in Children Using Primary Teeth Caries Experience	Pediatr Dent	T. S. Ghazal; N. K. Childers; S. M. Levy
9	Primary dentition (dmft) increment	2022	Acculturation and 4-year caries increment among children of foreign-born mothers in Sweden: a register-based cohort study.	BMC oral health	A. Granlund, A; F. C. Soares; A. Hjern; G. Dahllof; A. Julihn.
10	No differentiation of caries increment between primary and permanent dentition	1991	University of North Carolina caries risk assessment study. III. Multiple factors in caries prevalence	J Public Health Dent	R. C. Graves; J. R. Abernathy; J. A. Disney; J. W. Stamm; H. M. Bohannan
11	Primary dentition (dmft) increment	1991	The relationship between caries experience in the deciduous molars at 5 years and in first permanent molars of the same child at 7 years	Community Dent Health	M. M. Gray; M. D. Marchment; R. J. Anderson
12	No differentiation of caries increment between primary and permanent dentition	2015	High sucrose intake at 3 years of age is associated with increased salivary counts of mutans streptococci and lactobacilli, and with increased caries rate from 3 to 16 years of age	Caries Res	S. Karjalainen; M. Tolvanen; K. Pienihäkkinen; E. Söderling; H. Lagström; O. Simell; H. Niinikoski
13	No differentiation of caries increment between primary	2022	Pathways between Social Capital and Oral Health from Childhood to Adolescence.	Journal of dental research	Knorst, J. K., Brondani, B., Vettore, M. V., Hesse, D., Mendes, F. M., & Ardenghi, T. M.

	and permanent dentition				
14	Participants involved children > 71 months	2006	Employing dmft score as a risk predictor for caries development in the permanent teeth in Japanese primary school girls	J Oral Sci	M. Motohashi; H. Yamada; F. Genkai; H. Kato; T. Imai; S. Sato; A. Sugaya; M. Maeno
15	Primary dentition (dmft) increment	2007	A seven-year survival analysis of caries onset in primary second molars and permanent first molars in different caries risk groups determined at age two years	Acta Odontologica Scandinavica	P. Ollila; M. Larmas
16	Repeated dataset	2008	Long-term predictive value of salivary microbial diagnostic tests in children	Eur Arch Paediatr Dent	P. S. Ollila; M. A. Larmas
17	Participants involved children > 71 months at baseline	2015	Long-term caries development in schoolchildren and the role of educational status	Quintessence International	J. Schmoeckel; R. M. Santamaria; C. H. Splieth
18	Reporting at tooth level	2010	Modelling childhood caries using parametric competing risks survival analysis methods for clustered data	Caries Res	J. Stephenson; B. L. Chadwick; R. A. Playle; E. T. Treasure
19	No differentiation of caries increment between primary and permanent dentition	2011	Dietary Patterns and Oral Health Behaviours Associated with Caries Development from 4 to 7 Years of Age	Life (Basel, Switzerland)	C. C. Silva; S. Gavinha; S. Vilela; R. Rodrigues; M. C. Manso; M. Severo; C. Lopes, P. Melo
20	No differentiation of caries increment between primary	2019	Community-level predictors of Australian children's dental caries and injury	Australian Dental Journal	N. Stormon; P. J. Ford; R. Lalloo

	and permanent dentition				
21	Repeated dataset	2020	Family-Level Predictors of Australian Children's Dental Caries and Injuries	Pediatr Dent	N. Stormon; P. J. Ford; R. Lalloo
22	Participants involved children > 71 months at baseline	2021	Childhood Factors and Dental Caries in the Permanent Dentition: Findings of an 8-Year Study Under a Nationwide School Dental Service.	International Dental Journal	S. H. X. Tan; Y. Y. Teo; M. H. X. Tan; Gao, X. (2021).
