# Supplementary material for: Does Early Childhood Caries Increase Caries Development among School Children and Adolescents? A Systematic Review and Meta-Analysis
Source: Int J Environ Res Public Health. 2022 Oct 18;19(20):13459. doi: 10.3390/ijerph192013459 (PMC9603429; doi:10.3390/ijerph192013459)
Supplement: Supplementary file 1 [file ijerph-19-13459-s001.zip › File S3. Risk of bias of included studies.pdf]

Supplementary file S3. Risk of bias of included studies

		Bias due to confounding	Bias in selection of participants into the study	Bias in classification of exposures	Bias due to departures from intended exposures	Bias due to missing data	Bias in measurement of outcomes	Bias in selection of the reported result	Risk of overall bias
1	Alm (2012, SWE)	L	L	L	L	L	L	L	L
2	Al-Shalan (1997, USA)	S	L	L	L	S	L	L	S
3	Chankanka (2011, THA)	L	L	L	L	S	L	L	S
4	Cortellazzi (2013, BRA)	M	L	L	L	L	L	L	M
5	Du (2017, CHA)	S	L	L	L	L	L	L	S
6	Li & Wang (2002, CHA)	L	L	L	L	S	L	L	S
7	Saethre-Sundli (2020, NOR)	M	L	L	L	L	L	L	M
8	Skeie (2006, NOR)	S	L	L	L	M	L	L	S
9	Straetemans (1998, NLD)	S	L	L	L	S	L	L	S
10	Thibodeau (1999, USA)	S	L	L	M	S	L	L	S

L	Low risk of bias
M	Moderate risk of bias
S	Severe risk of bias

All studies were graded as low risk of bias in the domains (II) Bias in selection of participants into the study, (III) Bias in classification of exposure, and (VI) Bias in measurement of outcomes [1-10]. All studies were considered as low risk of bias in domain (VIII) Bias in the selection of the reported result [1-10], as they recruited their subjects in public clinics [1,2,8,9], hospitals [3], schools [4-6] of different districts, or approached all children of the location [9,10].

Five studies were graded with severe risk of bias in the domain (I) Bias due to confounding [2,5,8-10], as there were known confounding factors (sociodemographic factors) that were not measured or accounted for, which could have affected the true effect. Two studies were rated as of moderate risk of bias as they missed assessment of intermediate confounding factors, including diet, oral hygiene maintenance habits, presence of plaque, gingival inflammation and fluorosis [4,7].

For the domain (IV) Bias due to departures from intended exposure, nine studies were found of low risk as they either included fixed variables that would not change over time, or re-collected their data at each

follow-up [1-9]. Only one study found as moderate risk as the authors only used one baseline questionnaire to collect their data related to sociodemographic and oral health-related habits [10].

For the domain (V) Bias due to missing data, five studies were considered as serious risk of bias as there was a lack of reporting on the number of or impact of missing data [2,3,6,9,10]. Four studies were of low risk of bias as they had reported the percentage and reasons for missing data, and compared if there was any significant difference in the proportion of potential risk factors between the reviewed and withdrawn subjects with appropriate statistical tests [1,4,5,7]. One study was considered as of moderate risk of bias, as they have reported the reasons for dropout but did not evaluate if there was any significant difference in the distribution of potential risk factors between subjects who were reviewed and those lost to follow-up [8].

Only one study was graded as of low risk of overall bias as it was graded as low risk of bias in all seven domains [1]. Seven studies were graded as of serious risk of overall bias as they were graded as serious risk of bias in one or more domains [2,3,5,6,8-10]. The remaining studies were graded as of moderate risk of overall bias with more than one domain being graded as of moderate risk of bias [4,7].

Only one study was graded as of low risk of overall bias as it was graded as low risk of bias in all seven domains [1]. Seven studies were graded as of serious risk of overall bias as they were graded as serious risk of bias in one or more domains [2,3,5,6,8-10]. The remaining studies were graded as of moderate risk of overall bias with more than one domain being graded as of moderate risk of bias [4,7].

References

1. Alm, A.; Wendt, L.K.; Koch, G.; Birkhed, D.; Nilsson, M. Caries in adolescence - influence from early childhood. *Community Dent Oral Epidemiol* **2012**, *40*, 125-133, doi:10.1111/j.1600-0528.2011.00647.x.
2. al-Shalan, T.A.; Erickson, P.R.; Hardie, N.A. Primary incisor decay before age 4 as a risk factor for future dental caries. *Pediatr Dent* **1997**, *19*, 37-41.
3. Chankanka, O.; Cavanaugh, J.E.; Levy, S.M.; Marshall, T.A.; Warren, J.J.; Broffitt, B.; Kolker, J.L. Longitudinal associations between children's dental caries and risk factors. *J Public Health Dent* **2011**, *71*, 289-300, doi:10.1111/j.1752-7325.2011.00271.x.
4. Cortellazzi, K.L.; Tagliaferro, E.P.; Pereira, S.M.; Ambrosano, G.M.; Guerra, L.M.; de Vazquez, F.; de Meneghim, M.; Pereira, A.C. A cohort study of caries incidence and baseline socioeconomic, clinical and demographic variables: a Kaplan-Meier survival analysis. *Oral Health Prev Dent* **2013**, *11*, 349-358, doi:10.3290/j.ohpd.a30480.
5. Du, Q.; Yu, M.; Li, Y.; Du, H.; Gao, W.; Mei, H.; Liu, S. Permanent caries experience is associated with primary caries experience: a 7-year longitudinal study in China. *Community Dent Oral Epidemiol* **2017**, *45*, 43-48, doi:10.1111/cdoe.12257.
6. Li, Y.; Wang, W. Predicting caries in permanent teeth from caries in primary teeth: an eight-year cohort study. *J Dent Res* **2002**, *81*, 561-566, doi:10.1177/154405910208100812.
7. Saethre-Sundli, H.B.; Wang, N.J.; Wigen, T.I. Do enamel and dentine caries at 5 years of age predict caries development in newly erupted teeth? A prospective longitudinal study. *Acta Odontol Scand* **2020**, *78*, 509-514, doi:10.1080/00016357.2020.1739330.
8. Skeie, M.S.; Raadal, M.; Strand, G.V.; Espelid, I. The relationship between caries in the primary dentition at 5 years of age and permanent dentition at 10 years of age - a longitudinal study. *Int J Paediatr Dent* **2006**, *16*, 152-160, doi:10.1111/j.1365-263X.2006.00720.x.
9. Straetemans, M.M.; van Loveren, C.; de Soet, J.J.; de Graaff, J.; ten Cate, J.M. Colonization with mutans streptococci and lactobacilli and the caries experience of children after the age of five. *J Dent Res* **1998**, *77*, 1851-1855, doi:10.1177/00220345980770101301.
10. Thibodeau, E.A.; O'Sullivan, D.M. Salivary mutans streptococci and caries development in the primary and mixed dentitions of children. *Community Dent Oral Epidemiol* **1999**, *27*, 406-412, doi:10.1111/j.1600-0528.1999.tb02039.x.
